# The Prevalence of Internet Use as a Source of Information Among Patients With Hypertension

**DOI:** 10.7759/cureus.62730

**Published:** 2024-06-19

**Authors:** Wajeeha Saeed, Michael J Brockman, Melina Ortiz, Bhavi Trivedi, Sandesh Yohannan, Abdul Ahad Khan, Amish Parikh, Debabrata Mukherjee

**Affiliations:** 1 Internal Medicine, Texas Tech University Health Sciences Center El Paso, El Paso, USA; 2 Internal Medicine, Texas Tech University Health Sciences Center El Paso Paul L. Foster School of Medicine, El Paso, USA; 3 Internal Medicine, University of Texas Health Sciences Center at San Antonio, San Antonio, USA; 4 Internal Medicine, Memorial Medical Center, Las Cruces, USA; 5 Internal Medicine, Huntington Hospital, Pasadena, USA

**Keywords:** internet and source of health information, hispanic population, internet search, adult education, public health. hypertension

## Abstract

Background and objective

The incidence of hypertension is growing at an alarming rate globally. In the United States, nearly half of the adult population suffers from hypertension, a disease potentially associated with long-term dire consequences and comorbidities. While Internet access has proliferated, and free Internet-based education resources for healthy lifestyles have exponentially increased over the past two decades, little is known about whether Internet-based information can be or is used as a self-learning tool for hypertension management in a community setting. With almost no published data, if and to what degree Internet-based, self-directed learning tools are used for hypertension management needs to be assessed. In light of this, we aimed to evaluate the prevalence of Internet use as a source of information in patients with known diagnoses of hypertension who presented to our Internal Medicine clinic at Texas Tech University Health Sciences Center, El Paso.

Materials and methods

We conducted a single-center, cohort-based observational study at our teaching hospital's internal medicine clinic. A survey questionnaire was distributed to all adults aged more than 18 years with a known diagnosis of hypertension. Consent for participation was obtained from all participants. Of the total studied population, 93.6% (190/203) were of Hispanic descent. Moreover, 67.5% (137/203) identified as female. Of note, 22.7% (46/203) reported using the Internet to learn about hypertension. Internet users were younger, with a mean age of 61.4 years compared to 68.7 (p=0.02) years for non-Internet users, attended institutions of a higher grade of education, and mostly received information regarding hypertension from their families (91.3% vs 2.5%, p<0.001). While most patients used the Internet for making treatment decisions and were satisfied with their choices, more than a quarter felt confused and anxious after using Internet-based resources.

Results

Most patients in the study were found to not use the Internet as a resource tool for hypertension management (157/203; 77.3%). Among the 22.7% of patients who used the Internet for hypertension management, the most commonly utilized resource was Google.com, as an initial step to hypertension research (26/46, 58.6%, p<0.001), followed by multiple resources within a predetermined list on the provided survey (14/46, 30.4%). The survey also assessed the reasons for using Internet-based resources, with the primary reason being evaluating treatment options (19/46, 41.5%), followed by developing coping skills (13/46, 28.2%), and lastly aiding in decision-making (10/46, 21.5%).

Conclusions

Internet-based educational tools are mushrooming as the Internet is becoming more pervasive. This study shows that within this predominant Hispanic population, nearly one-quarter of patients with hypertension are using Internet-based, self-learning tools. This highlights a slow shift in medical education which providers have to be prepared for as patients will be using these tools as secondary information sources for medical decision-making more frequently going forward. Further studies need to be conducted to evaluate the current and longitudinal impact of these new information sources.

## Introduction

Hypertension is a major public health issue and a growing epidemic in the United States [1]. Nearly half of adults in the United States (47%, 116 million) have hypertension [2]. Individuals living with hypertension face multiple functional and psychological challenges, such as decreased functional activities due to end organ damage and depression [3-5]. Individuals with hypertension frequently require drastic lifestyle changes and medical intervention to mitigate adverse effects. Due to hypertension’s chronicity, patients may have to adhere to a strict lifelong medical regimen. Challenges often arise, especially in medically underserved populations, due to lack of continuity of care, financial issues, transportation challenges, etc. Management of hypertension, therefore, must encompass components of physiological and psychological treatment plans that take into account a patient’s social determinants of health as well. Ideally, patients need treatment management plans that empower them to manage their chronic illnesses as well as comply with their personal goals of care.

Many studies have evaluated tele-management strategies in treating hypertension [6-8]. To provide telehealth interventions effectively, healthcare professionals must recognize a patient's readiness to receive such interventions. This is especially important for older individuals who suffer from hypertension and are less inclined to use e-learning modules and take more time to learn than young adults with hypertension [9-12]. This can cause compounding problems because patients are often initially apprehensive about starting medications for treatment [13,14]. On top of needing to initiate sometimes lifelong therapies, patients would also have to occasionally learn cumbersome software for disease progression monitoring.

Tele-management also creates another, often forgotten dimension, i.e., the lack of technological infrastructure to support it. E-learning modules require an Internet-based device, either a computer, phone, or tablet, with a stable broadband Internet connection. Financial limitations can inhibit even the most eager of patients wanting to try Internet-based learning due to the high cost of cellular data plans and/or home Internet plans [15]. Technological infrastructure quality often parallels socioeconomic parameters, and hence it is important to have tele-management systems that are all-inclusive and robust so that healthcare disparities are not deepened [16-19]. Currently, there is a lack of research evaluating whether adult patients with hypertension are willing to use eHealth, Internet-based learning for the management of their disease, and, if so, evaluating barriers to providing such tools.

The Internet is recognized as an effective means for delivering important health information and support to the public [20,21]. As per data from 2006, 73% of American adults (32% of adults aged 65 years and older) were Internet users, and 20% of them explored online health information [22]. Though there is a significant amount of online health information being consumed, there is a lack of research on how this information is being used or if it is medically valid. There is a substantial corpus of management strategies and eHealth programs online about hypertension, an extremely prevalent disease in the United States. However, research evaluating if this Internet corpus is being utilized and if it is affecting health outcomes is limited. Coincidentally, Internet-based health programs have been successfully used for patients with other types of diseases and by their family members. They have been employed in the fields of cancer and AIDS, among support groups for people recovering from coronary artery bypass graft procedures, and osteoporosis [23-27].

Telehealth development also attracted substantial investment during the coronavirus disease 2019 (COVID-19) pandemic [28]. In 2020 and 2021, the United States federal government relaxed telehealth regulations, enabling increased investment, especially in rural communities. Though successful during the pandemic, questions remain on the long-term prospects of these investments and their future impact on patient care [29]. Internet applications have continually been shown to have a significant impact on communities, both local and global. Thus, it begs the question as to the potential impact Internet applications will play a part in the physician-patient relationship. As this is a challenging topic, there is a significant research gap in evaluating the optimal impact the Internet can have on a banal, often intangible disease such as hypertension. The speed of technological advancement and the ever-changing landscape after the COVID-19 pandemic have brought about significant challenges in studying telehealth’s impact [30,31]. More broadly, very little is known about how patients perceive and use the information gathered from the Internet.

Our study will attempt to give the first insights into assessing how many people are utilizing Internet-based sources for understanding and managing hypertension. Given where the study was conducted, it also provides a unique perspective on how Internet-based medical sources are viewed among minorities in the United States. Our study also provides a window into understanding the various challenges patients encounter while accessing the Internet, which can aid health professionals in formulating better eHealth programs. We aimed to assess the prevalence of Internet use in patients with a known diagnosis of hypertension who presented to our Internal Medicine clinic at Texas Tech University Health Sciences Center, El Paso, including data on resources used, ethnicity data, reasons for Internet use, and their outcomes, and the readiness of our population in using Internet and e-learning tools for exploring hypertension.

## Materials and methods

This was a cohort-based observational study using a questionnaire among patients with known diagnoses of hypertension consulting the Internal Medicine clinic at Texas Tech University Health Sciences Center, El Paso. All individuals included in this study were regular patients at the internal medicine primary care clinic. We enrolled all the subjects with a known diagnosis of hypertension, who were 18 years old or older, and treated at our Internal Medicine clinic, and the recruitment took place over a year. Subjects who did not consent to participate in the study, those who were not diagnosed with hypertension, and/or those younger than 18 years were excluded. We obtained approval from the Institutional Review Board at Texas Tech University Health Sciences Center El Paso (IRB#: E22096).

The study fundamentally relied on a survey questionnaire approved by the Institutional Review Board of Texas Tech University Health Sciences Center, El Paso. It was prepared in both English and Spanish to accommodate language barriers and patient preferences. The survey was created specifically for this study, and it had not been previously validated or standardized. Oral consent was taken from the patients to be enrolled in the study and for the administration of the one-time, anonymous survey distributed to each of the patients. The survey was administered to all enrolled patients (n=203). The survey questionnaire was distributed during the patients' regular scheduled clinic visits. To ensure the mitigation of biased responses, the survey was completed by the patients without any help from family members, nurses, or doctors. Since this was a one-time study, patients were expected to fill out and complete the questionnaire on the same day while they were in the clinic (see supplementary Appendix “A” for the administered survey questionnaire and collected variables).

Before conducting the statistical analysis, patients enrolled with a known past medical history of mental health conditions that impaired the reliability of response were excluded. The chi-square test of independence was used for survey questions involving categorical variable-based answers. Student t-test was used for survey questions involving continuous variable-based responses. A p-value of less than 0.05 was considered a statistically significant result compared to the null hypothesis.

## Results

Most patients enrolled in the study did not use the Internet as a resource or tool for the management of their hypertension (157/203; 77.3%). On the other hand, 22.7% (46/203) of patients used the Internet for hypertension management. The majority of patients enrolled in the study were found to be female, a ratio of nearly two-thirds female to one-third male (female: 137/203, 67.5%; male: 66/203, 32.5%). Regarding age, Internet-using patients were found to have a mean age of 61.4 ± 12.6 years while non-Internet users had a mean age of 68.76 ± 10.9 years (p=0.02). All patients studied were included in the analysis. The survey response rate was 100% for every section, without any missing data (203/203).

Most patients who actively used Internet-based resources were diagnosed with hypertension less than 10 years ago. Interestingly, when analyzing the percentage of patients who used Internet-based resources based on years since the initial hypertension diagnosis, a bimodal distribution was appreciated; 45.6% of Internet users were diagnosed with hypertension less than 10 years ago; 23.9% (11/46) of Internet resource users had a 10-20 year history of hypertension; 8.70% (4/46) of Internet resource users had a 20-30-year history of hypertension; finally, 21.7% (10/46) of Internet users had a history of 30 years or more of hypertension (p=0.032). As for gender, the patients who identified as male tended to use Internet-based resources more to manage their hypertension when compared to patients who identified as female (male: 20/46, 30.3%; female: 26/111, 19%). However, there were no significant differences among genders with regard to seeking the help of the Internet for hypertension management (p=0.07).

The unique perspective provided by the presented study pertains to its sample size, comprising mostly Hispanic patients. Of the total 203 patients in the study, 93.6% (190/203) identified as being of Hispanic descent. Of the 190 patients who identified as Hispanic, 42 (28.4%, 42/190) utilized Internet-based resources for self-directed management of their hypertension. The study included only 13 patients who did not identify as Hispanic, and hence limited interpretation could be made regarding the other identified ethnicities. When accounting for education, 67.4% (17/46) of Internet users had an elementary and/or middle school level of education. Of note, 91.3% (42/46) explained Internet-based resource utilization as a means to learn more about hypertension treatment. Expanding on this, 56.5% (26/46) of patients used the Internet for a multitude of reasons, such as hypertension treatment, medications, doctors, diet, etc; 2.17% (1/46) of patients used the Internet for support system information, and 23.9% (11/46) of patients used the Internet for general information about the diagnosis of hypertension.

**Figure 1:**
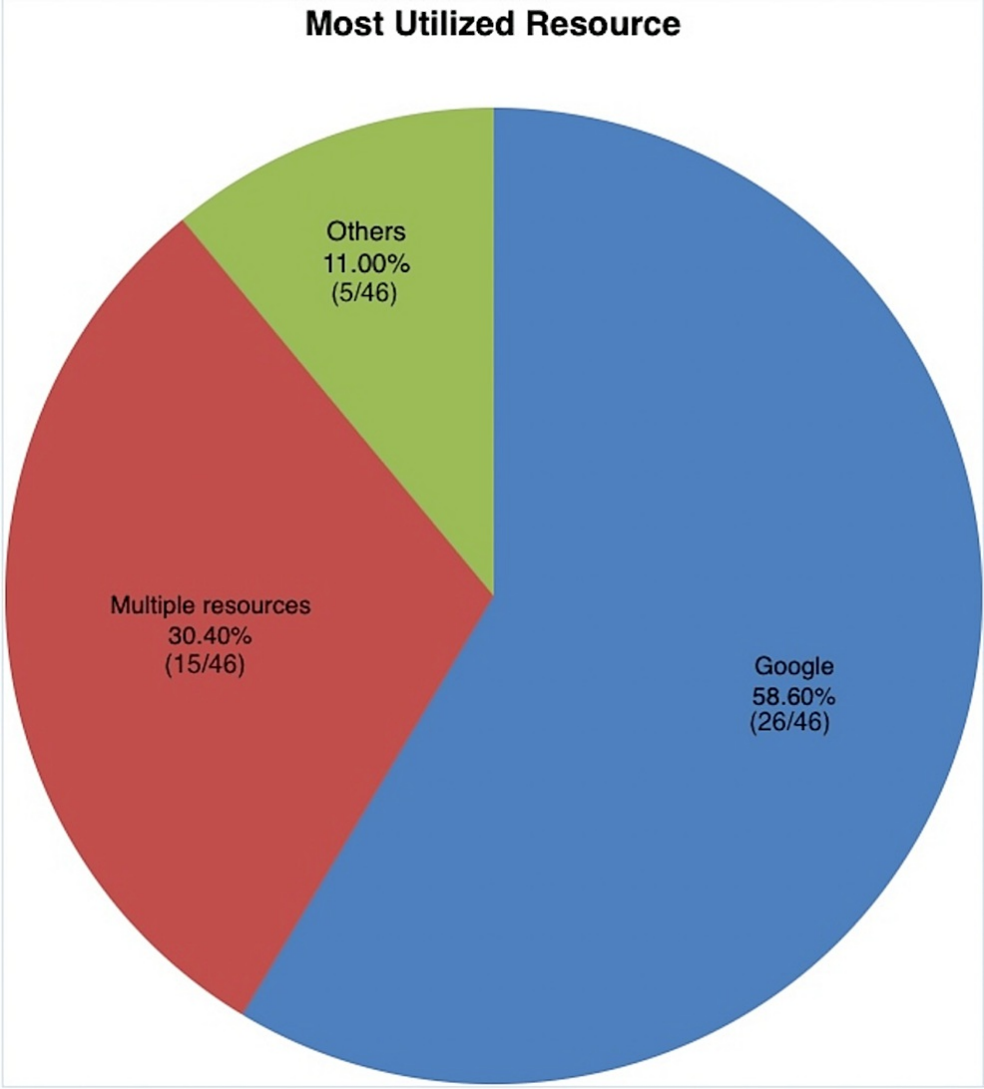
Tabulated frequency of the most utilized resources for hypertension research based on the questionnaire responses

The most utilized resource among the cohort was Google.com, as the initial step in hypertension research (26/46, 58.6%, p<0.001). Of note, 30.4% (14/46) were found to use multiple resources [Mayo, American Heart Association (AHA), WebMD, New England Journal of Medicine (NEJM), Wikipedia, Cable News Network (CNN), and Yahoo], with an additional 8.69% (4/46) reporting using websites other than those mentioned in the survey. Figure 1 visually represents the tabulated frequency of the most utilized resources for hypertension research.

**Figure 2:**
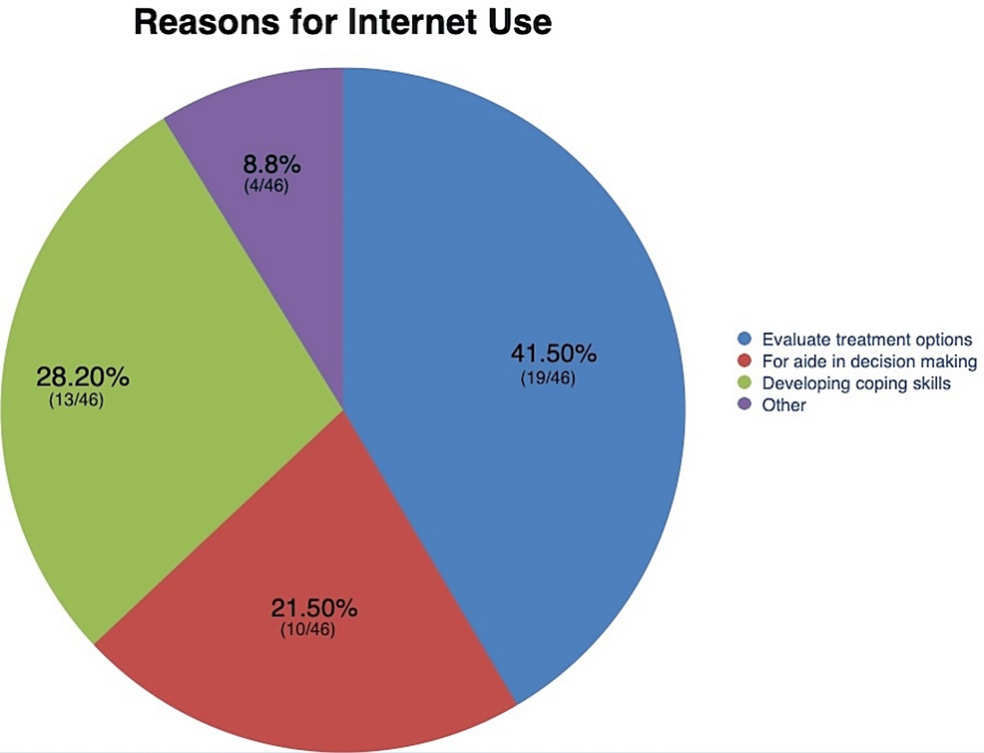
Tabulated frequency of the reasons for Internet usage for researching hypertension based on the questionnaire responses

The surveys provided also included queries as to why they were seeking further information on the Internet; 41.5% (19/46) of the Internet users reported that the objective of Internet usage was to evaluate treatment options; 21.5% (10/46) used to seek help in decision-making, and 28.2% (13/46) for developing coping skills. Figure 2 visually represents the tabulated frequency of the reasons for Internet usage for researching hypertension.

Of note, 76% of Internet users reported feeling hopeful and knowledgeable about their diagnosis of hypertension after using the Internet. Almost 24% (11/46) revealed feeling anxious, confused, and nervous about using the Internet for their hypertension management. Of note, 41 patients stated that they wanted to seek general information from the Internet about hypertension, 23 sought treatment options, 22 dietary information, 4.5% received information about alternative treatment strategies, 7.5% searched for information on support groups, 9% looked for medication-related inquiries, 9% looked up recent advances in medicine for the treatment of hypertension, and 4.5% used the Internet to search for doctors.

Regarding family and social support, 91.3% (42/46) of Internet users received help from their families in getting information about hypertension when compared to 2.55% (4/157) of non-Internet users (p<0.001). People who had more family support tended to use more Internet-based resources for the treatment of hypertension and reported achieving better control. After using the Internet for hypertension education, 39 out of 46 patients felt it was helpful (p<0.001), and seven patients remained indecisive; none of the patients felt it was not helpful. Most of the patients (39/46, 84.7%) discussed the findings on the Internet with their doctor (p<0.001). After discussion with their respective doctors, patients scored their level of knowledge to be accurate on a scale of 1 to 10, with 10 representing the most accurate level of knowledge (mean score: 7.87, median: 8, minimum: 5, and maximum: 10). Table 1 presents a detailed summary of the survey results.

**Table 1:** Survey results SD: standard deviation; AA: African American; LGA: last grade attended; FPS: family provided support

Variable	Internet users (n=46)	Non-Internet users (n=157)	P-value
Gender, n (%)			
Male	20 (30.3%)	46 (29.3%)	0.7
Female	26 (18.9%)	111 (70.7%)	
Age, years, mean ± SD	61.4 ± 12.6	68.8 ± 10.9	0.2
Race, n (%)			
White	1 (2.2%)	6 (3.8%)	0.31
Hispanic	42 (91.3%)	148 (94.2%)	
AA	1 (2.2%)	1 (0.6%)	
Other	2 (4.3%)	2 (1.3%)	
LGA, n (%)			
None	0 (0.0%)	12 (7.6%)	0.048
Elementary school	17 (36.9%)	71 (45.2%)	
Middle school	14 (3.0%)	28 (17.8%)	
High school	4 (8.7%)	23 (14.6%)	
College	11 (23.9%)	23 (14.6%)	
FPS, n (%)			
Yes	42 (91.3%)	4 (2.5%)	<0.001
No	4 (8.7%)	152 (96.8%)	

## Discussion

The United States has close to 311 million Internet users with over 90% of the American population having access to the Internet [29,32]. The ease of access and ubiquity of Internet access means that the United States ranks third in the world in terms of the percentage of the population active online [33]. The Internet has had a profound societal impact, especially in the educational field [34,35]. New-found interconnectedness has fostered the production of free resources that provide self-directed learning [36-38]. However, research on the effectiveness of these new tools is limited. In the field of healthcare, there are many resources for patients to educate themselves on common diseases, such as hypertension. However, the role and value of Internet-based, self-directed learning tools among patients with chronic comorbidities like hypertension have yet to be assessed. Even less is known when it comes to ethnic minorities and people with significant social determinants of health associated with more socioeconomic barriers [39].

Our study aimed to assess how patients with a diagnosis of hypertension educate themselves beyond the physician's examination room. Understanding the propensity for self-directed learning about one of the most common medical diseases, hypertension, can provide a window into not only how motivated patients are in going beyond medical management, but also evaluate the efficacy of Internet-based, self-directed resources. This study involved a unique population, as most of our enrollees were female and of Hispanic descent. Females and minorities have been consistently reported to have poorer health outcomes than their male and majority counterparts. Our results also raise unanswered questions that could be further explored, such as why Internet-based, self-directed learners were mostly women. While it could be attributed to the location of the study, it also raises another pertinent question: do minorities and females feel that physicians do not provide adequate instructions for managing their diseases? This may have prompted them to explore answers on their own on the Internet. It is also possible that Hispanic females are more prone to hypertension, which also warrants further investigations.

Regarding the age of the cohort, the patients studied were mostly 60 years or older; however, the study did include younger patients who tended to use Internet-based self-directed learning tools for hypertension. These trends are similar to the latest Internet adoption trends published by Pew Research Center [40]. In our study, we did not find significant differences in terms of gender in the usage of the Internet for hypertension-related self-directed learning. However, our study was limited due to a significantly smaller number of enrolled males.

When evaluating resources used, Google.com, unsurprisingly, was found to be the single most commonly accessed site for exploring information on hypertension, its disease management, medications, and the doctors in the area (58.6% of users). At the time this study was conducted, the Internet search giant accounted for over 90% of the global search market [41]. Even though Google is the dominant search engine, it brings into question the quality and accuracy of the content that the search engine provides. Being a search engine first, Google’s PageRank algorithm works by providing more popular websites over less popular websites in its search list, not most valid to least valid [42]. In other words, a Google query of “hypertension management” may not rank the AHA’s patient advocacy page on hypertension, but rather a social media website, due to the site’s popularity and search engine optimization. Tying into this concept is baseline education and utilization of Internet-based resources for hypertension management. Our study surprisingly did not show a major association between baseline education and Internet use. Hence we recommend that future studies explore the patient’s level of confidence in the reliability of the Internet-based resources based on their highest achieved education level.

Regardless of the confidence about the resource, age, or gender, it was found that 22.7% of the studied patients used Internet-based, self-directed learning tools for hypertension for their treatment options and decision-making on their treatment, and hence practitioners should consider this. Although it is reassuring that most patients (>70%) felt hopeful and knowledgeable, nearly 25% felt anxious and more confused after using these tools. Another aspect of Internet-based learning is the breadth of the available information. Using a simple search keyword can generate millions of articles, ranging from those dealing with broad aspects to the scientifically esoteric. This can lead to information overload, resulting in further confusion [33,43,44]. Given the variety of information patients can access on the Internet regarding hypertension, medical practitioners must assess if a patient plans on engaging in self-directed learning and educate them on the dangers of misinformation.

Another aspect that the findings of this study highlight is how the studied Hispanic population had a predilection to seek alternative treatment strategies and support groups for managing their hypertension. Though the reason behind this is not clear, possible explanations may highlight the importance of specific community-based hypertension management, and support group therapies like those in African Americans, where discussing hypertension treatment strategies in the barber shop led to a significant blood-pressure reduction when coupled with medication management in barbershops by specialty-trained pharmacists [45-47].

The adage that "medicine is an art and a science" applies especially to chronic illnesses. Though guideline-directed medical management can be initiated from the onset of the diagnosis, it does not guarantee medical compliance. Medical management, as well as family support, medication compliance, and physical activity in combination, are the best strategies for treating and eliminating the long-term effects of chronic illnesses such as hypertension [36,48,49]. As technology becomes more prevalent and ubiquitous in patients' lives, Internet-based, self-directed learning will become part of the treatment plan [50,51]. The goal of this study was to be a forerunner in evaluating the various aspects related to individuals in a predominantly minority border town using Internet-based, self-directed learning tools to manage their hypertension. Though this study answers many questions, it raises many more, such as the efficacy of the tool utilization, the confidence the patient base has in the tools, and the quality of information provided to the patient. Although the sample size was small, it is clear that Internet-based, self-directed learning tools enable patients to make medical decisions and modify their lifestyles. The impact of these decisions remains to be evaluated and investigated further.

Limitations

This was a single-center pilot study involving a population subtype. Larger studies are required to apply this study technique in a heterogeneous population. Although income status has been linked to healthcare outcomes, it does not always universally correlate to the ability to self-care. We did not look into the income status of the study participants. It can be evaluated in larger studies, specifically looking at hard outcomes.

## Conclusions

The Internet provides many resources for self-directed learning for hypertension management. Though these tools and resources are ubiquitous, often only requiring an Internet connection, they have never been studied at a community level. Internet adoption as a source for self-directed learning for hypertension was low in our study population, likely related to the fact the bulk of our cohort represented a predominantly underserved ethnic community with significantly higher socioeconomic barriers to healthcare access. Our study was conducted at a single center and predominantly involved a specific community sub-set. Similar trends need to be studied and explored in other minority communities. Our study highlights the importance of Internet-based resources in helping patients with informed decision-making for their hypertension management and the low prevalence of the usage of this resource in our cohort.

## Appendices

**Table 2:** Hypertension questionnaire

Questionnaire	
Date:	_____
Gender:	□ Male □ Female
Race:	□ Caucasian □ African American □ Hispanic □ Asian □ Other, please specify _______
Age:	_____
Year hypertension was diagnosed	_____
Last grade attended:	_____
Do you have access to the Internet?	□ Yes □ No
Do you use the Internet to get information about your hypertension:	□ Yes □ No
Do friends and family members provide you with information from the Internet?	□ Yes □ No
What are your feelings after you get the information from the Internet? Check all that applies:	□ Hopeful and positive □ Knowledgeable and in control of situation □ Increased anxiety □ Confused □ ﻿Nervous and upset □ ﻿Other, please specify: _____ □ Not applicable
Was the information you gathered from the Internet helpful to you?	□ Yes □ No, please explain why: ____ □ Not applicable
Do you discuss this information with your doctor?	□ Yes □ No, please explain why: ____ □ Not applicable
How accurate was the information that you were seeking on a scale of 1 to 10? 1 being least accurate and 10 being most accurate. Please check one box:	□ 1 □ 2 □ 3 □ 4 □ 5 □ 6 □ 7 □ 8 □ 9 □ 10 □ Not applicable
What type of information do you seek? Check all that applies:	□ General information about Hypertension □ ﻿﻿Treatment options for Hypertension □ ﻿﻿Recent advances and current research in Hypertension □ ﻿﻿Diet and nutrition related information □ ﻿﻿Information about Hypertension support groups □ ﻿﻿Alternative and complementary therapies □ ﻿﻿Medication related adverse effects □ ﻿﻿Information about different doctors in your area □ ﻿﻿Other, please specify: _____ □ ﻿﻿Not applicable
What websites do you visit? Check all that applies:	□ American Heart Association (AHA) □ ﻿﻿Mayo clinic □ ﻿﻿Medline Plus □ ﻿﻿WebMD. www.webmed.com □ ﻿﻿Google. www.health.google.com □ ﻿﻿Wikipedia □ ﻿﻿New England Journal of Medicine (NEJM) □ CNN □ ﻿﻿Yahoo. ﻿□ ﻿Other, please specify: _____ □ ﻿﻿Not applicable
Reason for using the Internet? Check all that applies:	□ To work with your doctor in the decision making process □ ﻿﻿To investigate treatment options □ ﻿﻿To help cope better with your condition □ ﻿﻿Other; Please Specify: _____ □ ﻿﻿Not applicable
